# Assessment of Turkish junior male physicians’ exposure to mobbing behavior

**DOI:** 10.3325/cmj.2012.53.357

**Published:** 2012-08

**Authors:** Bayram Sahin, Mehmet Cetin, Mesut Cimen, Nuri Yildiran

**Affiliations:** 1 Department of Healthcare Management, Faculty of Economics and Administrative Sciences, Hacettepe University, Ankara, Turkey; 2 Department of Military Health Services, Gulhane Military Medical Academy, Ankara, Turkey; 3 Department of Healthcare Management, Faculty of Health Sciences, Acibadem University, Istanbul, Turkey

## Abstract

**Aim:**

To determine the extent of Turkish junior male physicians’ exposure to mobbing behavior and its correlation with physicians' characteristics.

**Methods:**

The study included physicians recruited for compulsory military service in April 2009. No sampling method was used, questionnaires were delivered to all physicians, and 278 of 292 (95%) questionnaires were returned. We used Leymann Inventory of Psychological Terror including 45 items for data collection and structural equation model for data analysis.

**Results:**

A total of 87.7% of physicians experienced mobbing behavior. Physicians who worked more than 40 hours a week, single physicians, physicians working in university hospitals and private hospitals, and physicians who did not have occupational commitment were more exposed to mobbing (*P* < 0.05). Mobbing was not associated with specialty status, service period, age, and personality variables (*P* > 0.05). All goodness-of- fit indices of the model were acceptable (χ^2^ = 1.449, normed fit index = 0.955, Tucker Lewis index = 0.980, comparative fit index = 0.985, and root mean square error of approximation = 0.040).

**Conclusions:**

Workplace mobbing is a critical problem for junior male physicians in Turkey. We suggest an introduction of a reporting system and education activities for physicians in high-risk groups.

Mobbing has been shown to negatively affect the welfare and development of workers and organizations by increasing the rates of leave and absenteeism, lowering the morale, and causing anger, burnout, underperformance, and deterioration of corporate image and relationships among workers (1-5).

To minimize the number of cases, the issue of mobbing must first be thoroughly investigated (6). Although no definition has been generally accepted, mobbing usually refers to actions when someone is subjected to social isolation or exclusion, when their work and efforts are devaluated, and when they are threatened, worn down, or frustrated (7-10). The International Labour Organization (ILO) has defined the term as acting in unison against a coworker and exposing that individual to psychological harassment (11). The term mobbing has been used synonymously with suppression, attack, violence, bullying, psychological harassment, social isolation, threatening, and discrimination in the business life, and workplace trauma (12-14).

Mobbing has become a major problem in the health and other sectors (15-19). In the health sector, it can be increased by 24-hour service provision and intense work pace and correlates negatively with job satisfaction and performance, posing a threat to patients' safety (20-22).

In the ILO’s report from 2002, the health sector workers’ rate of exposure to mobbing in the USA has been sixteen times higher than in other sectors. More than half of the health personnel in different countries has in the previous year experienced at least one incident of physical or psychological violence (23,24). Although, there is no national study investigating the prevalence of mobbing in the health sector in Turkey, there have been some local studies focusing on the limited number of female nurses, indicating that mobbing is an important issue (25-28).

Although there are several studies on physicians’ exposure to mobbing, none of these investigated male physicians because it is generally thought that women are more often subjected to mobbing (29-33). However, negative and discomforting acts and interactions may not only be of a sexual nature or be solely directed toward women (9). This study aimed to determine the extent of junior male physicians’ exposure to mobbing behavior and to investigate its association with physicians’ characteristics.

## Participants and methods

### Sample

The research included 292 male physicians who started compulsory military service in the Ministry of Defense Samsun Military Medical Command in April 2009. All physicians gave informed consent and the participation was voluntary. A total of 278 (95%) questionnaires were returned.

### Data collection

To assess mobbing behavior, we used the Leymann Inventory of Psychological Terror (LIPT) scale, including 45 mobbing behavior types (7). The scale has 45 items classified into five dimensions: the items 1-11 refer to “behavior threatening communication,” the items 12-16 refer to “behavior threatening social contacts,” the items 17-31 refer to “behavior threatening personal reputation,” the items 32-40 refer to “behavior threatening occupational situation,” and the items 41-45 refer to “behavior threatening physical health.”

Frequency of exposure to mobbing is assessed with the following scores: 1 – every day, 2 – a few times a week, 3 – a few times a month, 4 – a few times a year, and 5 – never. Exposure to even one of the 45 types of behavior is enough to classify the participant as a victim of mobbing (7). The exposure to mobbing is assessed subjectively based on participants’ views and perceptions, but since the LIPT scale had been used in several previous studies (7,27,34,35) and its validity and reliability had been assessed, it was deemed suitable for the purposes of this study.

The participants who selected the answers between “every day” and “a few times a year” were united and classified as “people who were exposed to mobbing,” whereas the participants who selected “never” were classified as “people who were not exposed to mobbing” (1).

The reliability score for behavior threatening communication was 0.87, for behavior threatening social contacts 0.71, for behavior threatening personal reputation 0.90, for behavior threatening occupational situation 0.89, for behavior threatening physical health 0.74, and the general reliability value was 0.95.

### Data analysis

To analyze the questionnaire data, SPSS, version 14.0 (SPSS Inc., Chicago, IL, USA) and AMOS, version 6.0 (Amos Development Corporation, Spring House, PA, USA), software programs were used. While descriptive statistics was used to present the percentage of participants’ exposure to mobbing behavior, structural equation model (SEM) was used as a multivariate statistical method to determine the factors (physicians characteristics) affecting the level of exposure to mobbing behavior (36-38).

### Structural equation model

SEM encompasses two major components: measurement and structural model. The measurement model establishes the relationships between latent (unobserved) variables and multiple observable items. This is the confirmatory factor analysis portion of a model. The structural model tests a set of hypothesized associations between two or more variables. It includes a set of paths (regression coefficients) or correlations between the various measured and unmeasured variables in the overall model (36-41).

In this research, in order to obtain the model that best explains whether there was a significant relation between the level of physicians’ exposure to mobbing behavior and their characteristics, two models were developed and tested. The first used 19 variables, 8 of which were independent variables reflecting the physicians’ sociodemographic characteristics (work place [1 = Ministry of Health hospital, 2 = university hospital, 3 = private hospital], marital status [1 = married, 2 = single], specialty status [1 = practitioner, 2 = specialist physician], number of working hours per week [1 = 40 hours, 2 = 41-56 hours, 3 = 57 hours and more], age [years], duration of work [years], occupational commitment [1 = yes, 2 = no], and personality [1 = ambitious, 2 = emotional, 3 = passive]). Five variables (behavior threatening communication, behavior threatening social contacts, behavior threatening personal reputation, behavior threatening occupational situation, and behavior threatening physical health) were observed indicator variables and another five were unexplained indicators. Finally, the last variable was mobbing, which was the latent dependent variable. The second, revised, model used only four of socio-demographic characteristics that were found significant.

There are various indices to evaluate the goodness of fit of SEM. Most commonly used are χ^2^ value, comparative fit index (CFI), normed fit index (NFI), Tucker-Lewis index (TLI), and root-mean-square error approximation (RMSEA) (40).

A high χ^2^ value indicates that the model fit is poor; as it is dependent on sample size and both observed and expected covariance matrix, it has only a limited use. As the sample size increases with the residual covariance matrix, the χ^2^ value and the probability for rejecting the model will increase (42). Frequently used CFI, NFI, and TLI fitness measures have values between 0 and 1, with values closer to 1 indicating better fitness (36). For RMSEA, it has been argued that values less than or equal to 0.05 indicate excellent fit, between 0.08 and 0.10 indicate acceptable fit, and higher values indicate unacceptable fit (41).

## Results

The mean age and service period of male physicians were 32.4 ± 2.5 years and 5.3 years ±2.9, respectively (Table 1). More than a half of the physicians (72.4%) were serving in hospitals affiliated to the Ministry of Health and the remaining were working in university and private hospitals. All of them were civilian physicians doing military service, rather than military personnel. A total of 18.3% had an administrative position such as head physician and vice head physician, 68.1% were married, 71.9% were specialist physicians, 71.7% worked more than 40 hours a week, 67% defined themselves as ambitious and hardworking, and 10.8% did not like medical profession (Table 1).

**Table 1:** Personal characteristics of Turkish male physicians doing military service

Personal characteristics	No. (%) of physicians
**Age in years (mean**±standard deviation**)**	32.4 ± 2.5
**Service period in years (mean**±standard deviation**)**	5.3 ± 2.9
**Administrative position:**	
yes	51 (18.3)
no	228 (81.7)
**Place of work:**	
Ministry of Health hospitals	202 (72.4)
university hospitals	42 (15.1)
private hospitals	35 (12.5)
**Marital status:**	
married	190 (68.1)
single	89 (31.9)
**Specialty:**	
practitioner	78 (28.1)
specialist	200 (71.9)
**Weekly working hours:**	
40 h	79 (28.3)
41-56 h	126 (45.2)
57 h and more	74 (26.5)
**Personality:**	
ambitious and hardworking	188 (67.4)
emotional	68 (24.4)
passive	23 (8.2)
**Occupational commitment:**	
yes	248 (89.2)
no	30 (10.8)

**Figure 1:**
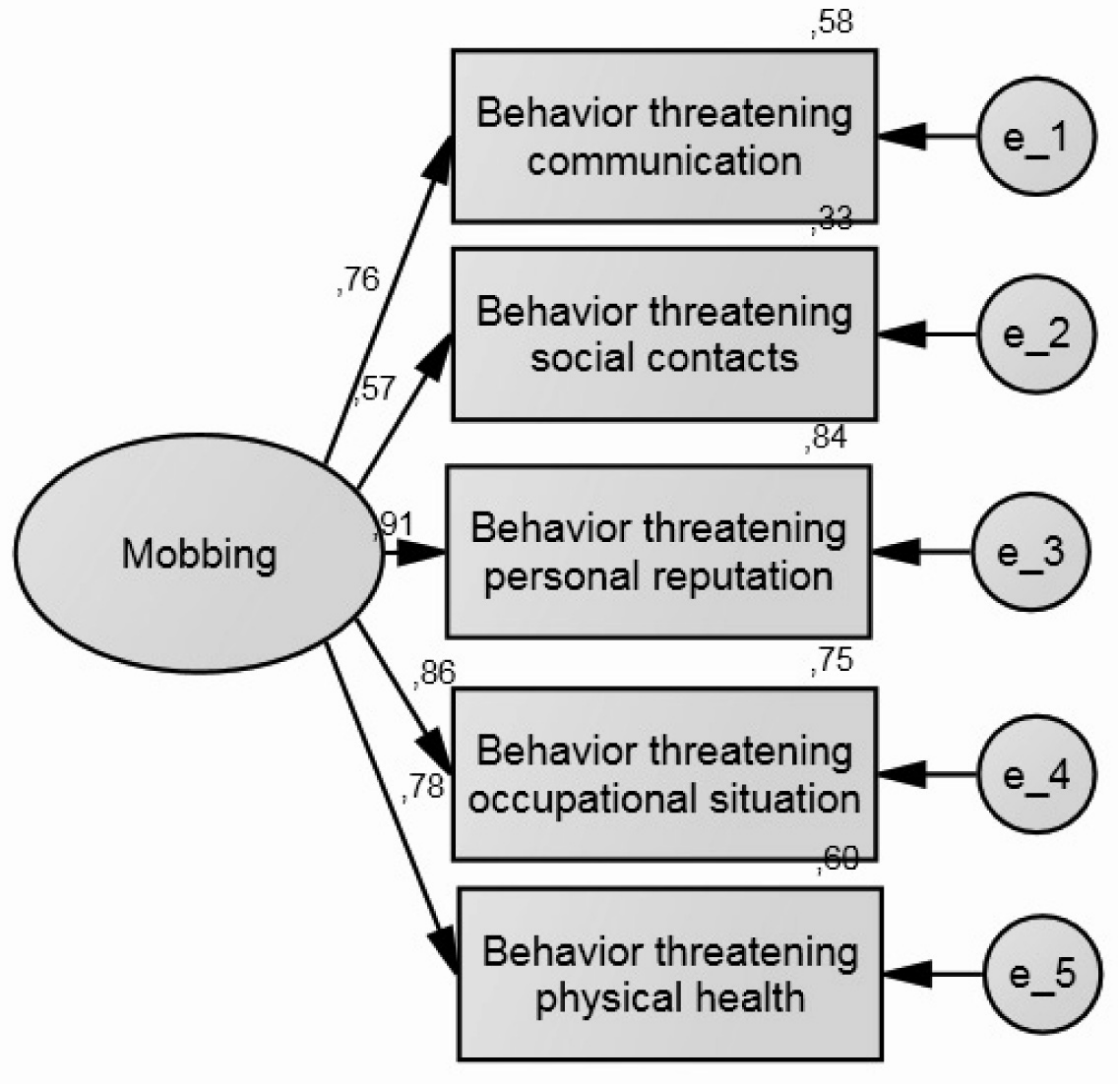
The measurement model identifying physicians’ exposure to mobbing behavior. χ^2^/df = 12.865/5 = 2.573; normed fit index = 0.984; Tucker Lewis index = 0.980; comparative fit index = 0.990; and root mean square error of approximation = 0.075. Rectangles represent independent and indicator variables and ellipses represent latent variables. The arrows from latent variables to indicators show regression and indicator weights. The error for each variable is represented by the arrow pointing to the variable and “e” in the circle. These errors correspond to the errors in the indicator variables.

A total of 87.7% of 278 participants were exposed to at least one of the 45 types of mobbing behavior defined in the questionnaire: 79.5%, reported “behavior threatening communication,” 64.7% reported “behavior threatening personal reputation,” 57.2% reported “behavior threatening occupational situation,” 48.9% reported “behavior threatening social contacts,” and 31.3% reported “behavior threatening physical health” (Table 2) (Figure 1).

**Table 2:** Mobbing behavior experienced by male physicians

Mobbing behaviors	Mean	Standard deviation	Non-victims			victims		
			No.		%		No.	%
**Behavior threatening communication**	4.4	0.6	57		20.5		221	79.5
1. The aggressor or mobber gives the victim no possibility to communicate	4.1	1.1	136		48.9		142	51.1
2. The victim is silenced or continuously interrupted	4.1	1.1	132		47.5		146	52.5
3. Colleagues prevent the victim to communicate	4.2	0.9	138		49.6		140	50.4
4. Colleagues scream and shout at the victim	4.6	0.8	201		72.3		77	27.7
5. The victim suffers verbal attacks regarding work assignments	4.3	0.9	142		51.1		136	48.9
6. The victim suffers verbal attacks regarding her/his personal life	4.7	0.7	216		77.7		62	22.3
7. The victim is terrorized by means of phone calls	4.3	1.1	173		62.2		105	37.8
8. The victim suffers verbal threats	4.6	0.7	204		73.4		74	26.6
9. The victim suffers written threats	4.9	0.4	255		91.7		23	8.3
10. People at work refuse to make any contact with the victim	4.5	0.8	173		62.2		105	37.8
11. The victim's presence is ignored	4.5	0.8	172		61.9		106	38.1
**Behavior threatening social contacts**	4.7	0.5	142		51.1		136	48.9
12. The aggressor does not talk to the victim	4.8	0.5	223		80.2		55	19.8
13. The victim is forbidden to talk to the aggressor	4.8	0.6	232		83.5		46	16.5
14. The victim is isolated in a room far away from others	4.3	1.4	208		74.8		70	25.2
15. Colleagues are forbidden to talk to the victim	4.9	0.5	261		93.9		17	6.1
16. The physical presence of the victim is denied	4.7	0.7	220		79.1		58	20.9
**Behavior threatening personal reputation**								
17. Slanders and lies about the victim are used at work	4.4	0.8	158		56.8		120	43.2
18. The victim is gossiped	4.4	0.8	159		57.2		119	42.8
19. The victim is ridiculed	4.7	0.6	222		79.9		56	20.1
20. The victim is said to have a mental illness	4.9	0.4	266		95.7		12	4.3
21. The aggressor tries to make the victim go through psychiatric exams	4.9	0.3	266		95.7		12	4.3
22. The victim is supposed to be ill	4.8	0.5	242		87.1		36	12.9
23. The victim's voice, gestures, and way of moving are imitated	4.8	0.6	230		82.7		48	17.3
24. The victim suffers verbal attacks regarding her/his political and religious beliefs	4.8	0.5	234		84.2		44	15.8
25. People at work make fun of the victim's personal life	4.8	0.5	238		85.6		40	14.4
26. People at work make fun about the ethnic origin or nationality of the victim	4.9	0.4	262		94.2		16	5.8
27. The victim is forced to do humiliating jobs	4.8	0.6	234		84.2		44	15.8
28. The victim is controlled and his/her job performance is tracked for those with bad intentions	4.7	0.6	208		74.8		70	25.2
29. Victim's decisions are questioned	4.5	0.7	177		63.7		101	36.3
30. The victim is reviled using obscene or degrading terms	4.9	0.5	260		93.5		18	6.5
31. The victim is sexually harassed	4.9	0.4	260		93.5		18	6.5
**Behavior threatening occupational situation**	4.7	0.5		119	42.8	159		57.2
32. The victim is not given any work assignments at all	4.7	0.7		219	78.8	59		21.2
33. The victim is deprived of any activity when being at work	4.8	0.5		236	84.9	42		15.1
34. The victim is given meaningless work assignments	4.6	0.7		188	67.6	90		32.4
35. The victim is given work assignments far below her/his capacity	4.4	0.9		172	61.9	106		38.1
36. The victim is continuously given new work assignments	4.7	0.6		224	80.6	54		19.4
37. The victim is given humiliating work assignments	4.6	0.8		210	75.5	68		24.5
38. The victim is given difficult work assignments far above her/his capacity	4.7	0.7		228	82.0	50		18.0
39. The victim is deliberately forced to spend big sums of money	4.7	0.7		209	75.2	69		24.8
40. Accidents are caused in the victim's workplace or home	4.9	0.4		261	93.9	17		6.1
**Behavior threatening physical health**	4.8	0.4		191	68.7	87		31.3
41. The victim is given dangerous work assignments	4.6	0.9		207	74.5	71		25.5
42. The victim is physically threatened	4.8	0.5		245	88.1	33		11.9
43. The victim is physically attacked as a threat	4.9	0.5		251	90.3	27		9.7
44. The victim is physically attacked with serious consequences for his/her health	4.9	0.3		264	95.0	14		5.0
45. The victim is sexually attacked	5.0	0.3		272	97.8	6		2.2
**Overall mobbing**				34	12.3	244		87.7

**Figure 2:**
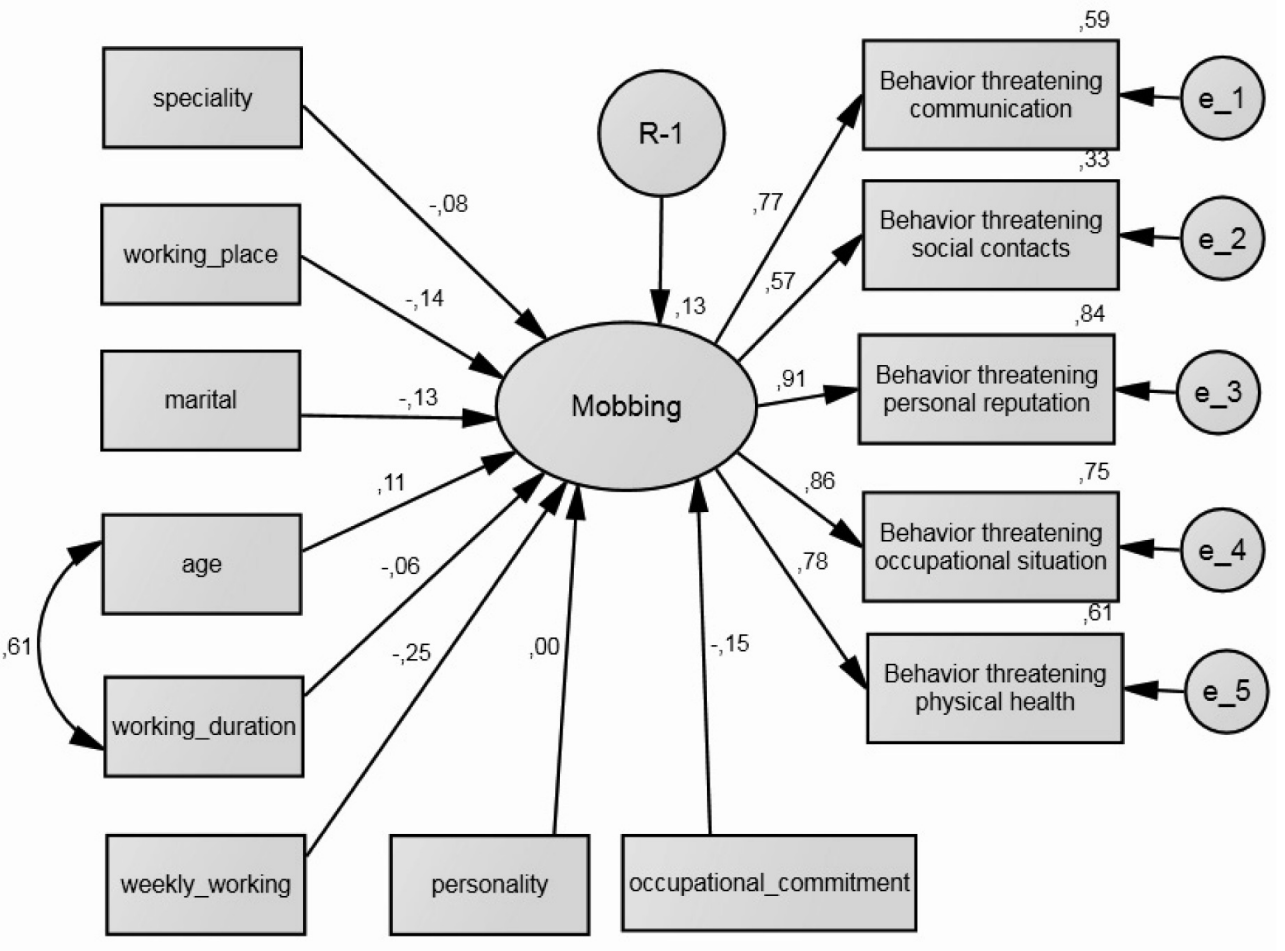
The initial structural equation model identifying determinants of the physicians’ exposure to mobbing behavior. χ^2^/df = 282.611/64 = 4.416; normed fit index = 0.771; Tucker Lewis index = 0.728; comparative fit index = 0.809; root mean square error of approximation = 0.111. Rectangles represent independent and indicator variables and ellipses represent latent variables. The arrows from latent variables to indicators show regression and indicator weights. The error for each variable is represented by the arrow pointing to the variable and “e” in the circle. These errors correspond to the errors in the indicator variables.

The measurement model shows the validity of the LIPT scale used to determine the level of male physicians’ exposure to mobbing behavior (Figure 1). To show whether the suggested model was compatible with the research data, a confirmatory factor analysis was conducted. As the standardized regression parameter values of five dimensions were higher than 0.50 (between 0.573-0.915), and all dimensions significantly related to the latent variable for mobbing (*P* < 0.001), it was accepted that the suggested measurement model was compatible with the research data and it had convergent validity. Furthermore, confirmatory factor analysis (Table 3) showed that mobbing behavior threatening occupational status and social contacts had more weight to explain the latent variable of mobbing. In other words, these dimensions best explained the variance in latent variable. On the other hand, the goodness-of-fit criteria of the measurement model were acceptable, except for the χ^2^ value, which was sensitive to the size of the sample (χ^2^ = 2.573, NFI = 0.984, TLI = 0.980, CFI = 0.990, and RMSEA = 0.075) (Figure 2).

**Table 3:** Regression coefficients of the measurement model identifying male physicians’ exposure to mobbing behavior

	Standardized regression coefficients	Standard error	*t* test	*P*
Behavior threatening communication	0.763	0.051	14.609	<0.001
Behavior threatening social contacts	0.573	0.062	9.474	<0.001
Behavior threatening personal reputation	0.915	0.048	16.260	<0.001
Behavior threatening occupational situation	0.864	0.063	15.032	<0.001
Behavior threatening physical health	0.777	0.049	13.368	<0.001

The next step was to determine which independent variables influenced the extent of exposure to mobbing (Figure 2). In the initial structural equation model, eight personal characteristics were used as the explanatory variable: specialty status, working place, marital status, age, service period, weekly working hours, personality and occupational commitment. Working place (*t* = -2.226; *P* = 0.023), marital status (*t* = -2.103; *P* = 0.035), weekly working hours (*t* = -4.007; *P* = <0.001), and occupational commitment (*t* = -2.504; *P* = 0.012) significantly affected the extent of exposure to mobbing (Table 4), while specialty status, service period, age, and personality variables did not (*P* > 0.05). Moreover, the goodness-of-fit criteria of the suggested initial structural equation model pointed out that the model was not within acceptable limits (*χ^2^* = 4.416, NFI = 0.771, TLI = 0.728, CFI = 0.809, and RMSEA = 0.111).

**Table 4:** Regression coefficients of the initial structural equation model estimating determinants of the physicians’ exposure to mobbing behavior

	Standardized regression coefficients	Standard error	*t* test	*P*
Working place	-0.137	0.060	-2.266	0.023
Marital status	-0.134	0.061	-2.103	0.035
Specialty status	-0.084	0.077	-1.096	0.273
Service period	-0.061	0.013	-.753	0.451
Age	0.109	0.015	1.272	0.203
Weekly working hours	-0.246	0.037	-4.007	<0.001
Personality	-0.001	0.042	-.013	0.989
Occupational commitment	-0.152	0.088	-2.504	0.012

**Figure 3:**
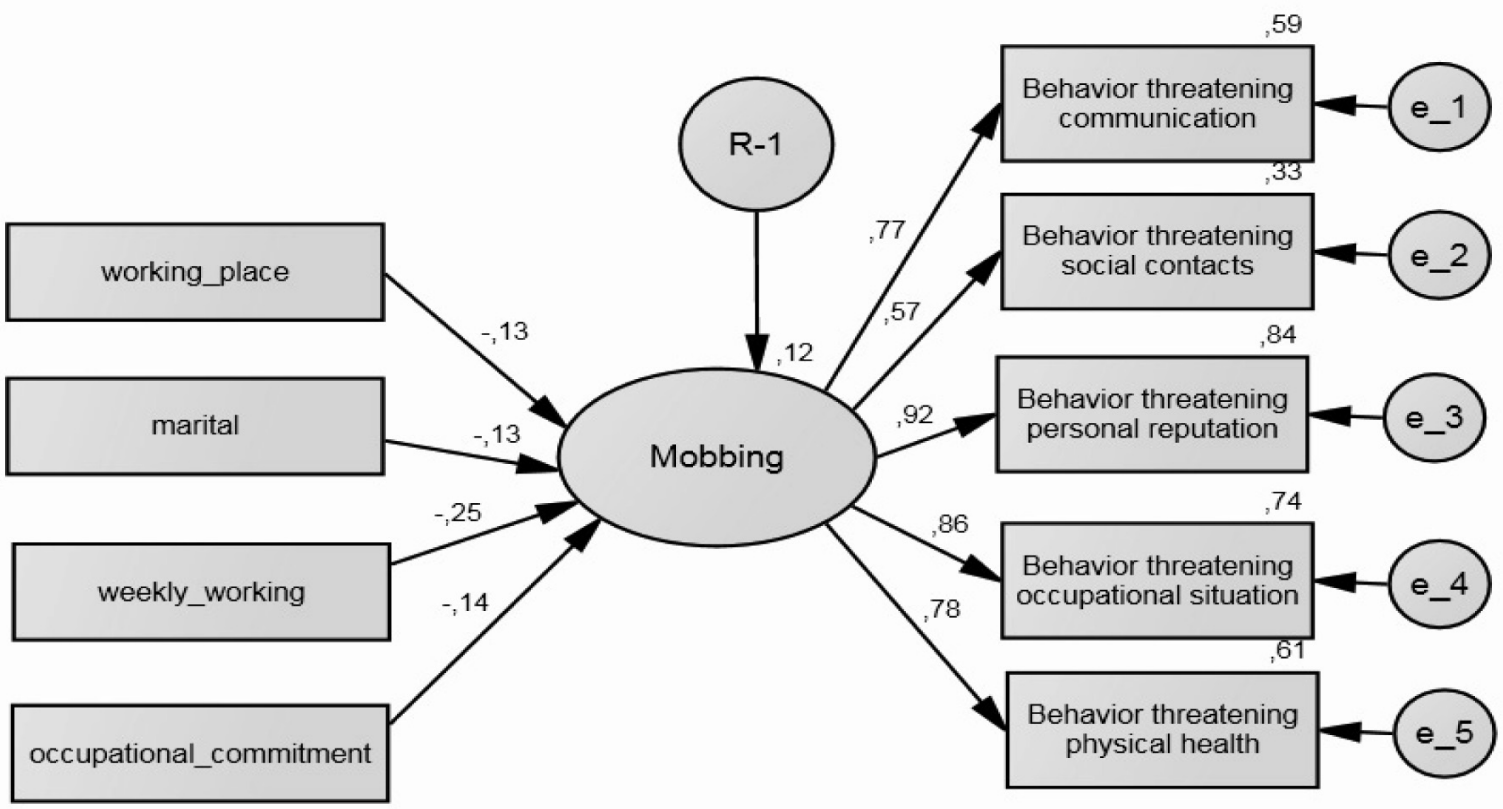
The revised structural equation model identifying determinants of the physicians’ exposure to mobbing behavior. χ^2^/df = 39.135/27 = 1.449; normed fit index = 0.955; Tucker Lewis index = 0.980; comparative fit index = 0.985; root mean square error of approximation = 0.040.

The four independent variables that were proven to be non-significant in the initial structural equation model were excluded and a revised new structural equation model was constituted (Figure 3) to achieve higher goodness-of-fit values and higher compatibility of the model to the data (Table 5).

**Table 5:** Regression coefficients of the revised structural equation model estimating determinants of the physicians’ exposure to mobbing behavior

	Standardized regression coefficients	Standard error	*t* test	*P*
Weekly working hours	-0.252	0.037	-4.135	<0.001
Working place	-0.132	0.059	-2.207	0.027
Marital status	-0.132	0.057	-2.216	0.027
Occupational commitment	-0.141	0.086	-2.362	0.018

The revised and improved model showed that the four independent variables (weekly working hours, marital status, working place, and occupational commitment) had a significant influence on mobbing (Table 5). Physicians who worked more than 40 hours a week, single physicians, and physicians working in university hospitals and private hospitals were more likely to be exposed to mobbing. A negative relationship was found between occupational commitment and exposure to mobbing – physicians who did not have occupational commitment were more exposed to mobbing.

The goodness-of-fit indices of the revised structural equation model increased significantly in comparison with the initial structural equation model, and all indices were within acceptable limits (*χ^2^* = 1.449, NFI = 0.955, TLI = 0.980, CFI = 0.985, and RMSEA = 0.040). Therefore, as goodness-of-fit values improved, the model was adopted as the final model. Total explanatory coefficient of the model was 12%. In other words, four independent variables that were found to be significant in the revised structural equation model were able to clarify only 12% of the variance of physicians’ level of exposure to mobbing (Table 5).

## Discussion

Our study showed that almost nine of ten physicians had a mobbing experience in the previous year and the frequency of mobbing exposure was higher than in other studies (2,11,19,22,24,43-45). These differences may be a result of the use of different mobbing definitions, scales, recall periods (46,47), settings (48-50), and participants (physicians or nurses) (28,43,51-55).

In this study, the most common mobbing behavior was “behavior threatening communication” and the least common was “behavior threatening physical health.” “Behavior threatening communication” was the most common mobbing behavior in other studies on health workers (16,19,27,43,53,56).

Another interesting finding was that the extent of exposure to mobbing was higher in the university hospitals and private hospitals than in the Ministry of Health hospitals, similar as in the study by Sahin and Dundar (27). This could be attributed to the greater complexity of university hospitals and a more stressful working environment (45,47).

While most of the mobbing victims in this study were single, in the study by Kowalczuk et al (57) they were mostly married. Greater exposure of single physicians to mobbing can be explained by their younger age and lack of experience. Also, married physicians can be positively discriminated in terms of lower work load, especially working the night shifts. We also found that physicians working more than 40 hours were exposed to mobbing more than those working less than 40 hours. This could be attributed to the hectic work environment that paves the way for mobbing, or working more than others might be regarded as unfair and as mobbing behavior. Finally, we also found that physicians who did not show occupational commitment complained about mobbing behavior more than those showing occupational commitment. This can be explained by a greater exposure to mobbing of physicians without occupational commitment or loss of occupational commitment in physicians exposed to mobbing.

As this study is a descriptive study of physicians doing compulsory military service, the results cannot be generalized to all male physicians. Also, due to study design we were not able to determine the causal relationship between the variables. Also, a recall bias might have occurred because physicians had to report their experiences in the past year and some of the physicians may not have wanted to share their personal experience. Cowie et al (58) found that questionnaire formats were not sufficient in investigating mobbing. Another limitation was that four variables (working place, marital status, weekly working hours and occupational commitment) that had significant effects on the frequency of exposure to mobbing explained only 12% of variance. The advantage of this study is the use of SEM, which is a multivariate statistical analysis method.

In conclusion, we found that the physicians working in university or private hospitals, working more than 40 hours in a week, single physicians and those without professional commitment were more exposed to mobbing. In order to take proper preventive measures against mobbing, its exact causes must be determined and legal regulations should be introduced. Health care providers should also be informed about mobbing and their legal rights. Thus, hospital department heads must monitor the development of mobbing behavior, come up with solutions by making a risk analysis, and provide an environment in which employees are able to express their complaints.
